# Emergence of invasive candidiasis with multiple *Candida* species exhibiting azole and echinocandin resistance

**DOI:** 10.3389/fmicb.2025.1550894

**Published:** 2025-03-25

**Authors:** Si-Jia Huang, Yi-Hui Song, Geng Lv, Jin-Yan Liu, Jun-Tao Zhao, Lu-Ling Wang, Ming-Jie Xiang

**Affiliations:** ^1^Department of Laboratory Medicine, Ruijin Hospital Luwan Branch, Shanghai Jiao Tong University School of Medicine, Shanghai, China; ^2^Department of Laboratory Medicine, Ruijin Hospital, Shanghai Jiao Tong University School of Medicine, Shanghai, China; ^3^The Shanghai Institute of Hypertension, Ruijin Hospital, Shanghai Jiao Tong University School of Medicine, Shanghai, China

**Keywords:** invasive candidiasis, *Candida glabrata*, *Candida norvegensis*, *Candida tropicalis*, antifungal susceptibility testing, azole resistance, echinocandin resistance

## Abstract

**Background:**

Invasive candidiasis (IC) is an increasingly common, expensive, and potentially fatal infection. However, IC caused by multiple *Candida* species is rarely reported in China. Herein, we revealed a complex IC caused by multiple *Candida* species, comprising the rare *C. norvegensis*, *C. albicans*, *C. glabrata*, and *C. tropicalis*. The resistance mechanism of azole and echinocandin resistance were explored further.

**Methods:**

The isolates were confirmed using internal transcribed spacer (ITS) sequencing. The resistance mechanisms were investigated using PCR-based sequencing, quantitative real-time reverse transcription PCR, and rhodamine 6G efflux quantification.

**Results:**

Antifungal susceptibility testing showed this complex infection was associated with cross-resistance to azole and echinocandin drugs. For *C. glabrata*, the acquired echinocandin resistance was likely caused by a novel mutational pattern (1,3-beta-D-glucan synthase subunits FKS1-S629P and FKS2-W1497stop) while the acquired azole resistance in *C. glabrata* RJ05 was related to complex mechanisms including enhanced efflux activity, pleiotropic drug resistance 1 (PDR1) mutation, and increased expression of *Candida* drug resistance 1 (CDR1) and CDR2. Additionally, the azole resistance of *C. tropicalis* was caused by two lanosterol 14-alpha demethylase (ERG11) mutations: Y132F and S154F.

**Conclusion:**

Our study revealed a case of clinically complex, multiple *Candida* invasive infections, further uncovering the resistance mechanisms to azoles and echinocandins. These findings provide valuable references for the diagnosis and treatment of invasive candidiasis (IC) in clinical practice.

## 1 Introduction

*Candida* species can colonize multiple parts of the human body, causing diseases when the body’s immune system is impaired or autoimmune barriers are disrupted (Giannella et al., 2024; Iliev et al., 2024). Invasive fungal diseases (IFDs) have become a major threat to public health, with high incidence and fatality rates, resulting in approximately 3.8 million patient deaths each year (Bassetti et al., 2017; Lass-Flörl et al., 2024; Sedik et al., 2024). Among them, invasive candidiasis (IC) is an increasingly common, expensive, and potentially fatal infection. Previously, IC was mainly caused by *Candida albicans*. However, in the past decade, the contribution of non*-C. albicans Candida* species (NCACs) to IC has increased (Bays et al., 2024; Salimi et al., 2024). More importantly, infections by rare *Candida* species, such as *C. norvegensis*, have displayed a rising trend over recent years (Pinho et al., 2024).

Antifungal resistance constitutes an important issue in the treatment of *Candida* infections. Triazoles like fluconazole (FLU), voriconazole (VRC), itraconazole (ITR), and posaconazole (POS) are the most commonly used antifungals. These drugs via bind to and inhibit the key ergosterol biosynthesis pathway enzyme, 14-α-demethylase, which is encoded by *ERG11*. However, resistance to azoles has become very common (Meneghello et al., 2024). To combat this, it is recommended to use echinocandins, including caspofungin (CAS), micafungin (MIF), and anidulafungin (ANF), as first-line IC treatments, because they target the catalytic subunit of β-1,3-glucan synthase (encoded by *Candida* gene *FKS1*, except in *C. glabrata*, in which it is encoded by *FKS1* and *FKS2*)(Pappas et al., 2016). This enzyme is crucial for the biosynthesis of β-1,3-glucan, a component of the cell wall of fungi. However, the emergence of echinocandin resistance is a concerning trend, with *C. glabrata* isolates showing resistance rates of 10.3% in a cancer center in United States (Farmakiotis et al., 2014). However, in China, the resistance rate is below 1% (Hou et al., 2017; Song et al., 2020).

Azole resistance among *Candida* spp. results from ATP-binding cassette (ABC) family multidrug transporter overexpression, including that of *CDR1*, *CDR2*, and *SNQ2* (Marie and White, 2009; Abbes et al., 2013). These transporters are regulated by the zinc finger transcription factor *PDR1*. Therefore, functional mutations in *PDR1* can lead to reduced susceptibility to azoles (Ferrari et al., 2009). Meanwhile, the overexpression of drug targets (lanosterol 14α-demethylase, encoded by *ERG11*) and mutations are also common (Hull et al., 2012; Nguyen et al., 2024; Tanwar et al., 2024). What’s more, alternative mechanisms, like biofilm formation and mitochondrial defects, have also been reported recently (Ferrari et al., 2011; Nguyen et al., 2024). In contrast, the mechanisms underlying echinocandin resistance are relatively straightforward, primarily involving mutations in the hotspot regions of *FKS1* and *FKS2* encoding subunits of the 1,3-β-D-glucan synthase complex (Zimbeck et al., 2010; Arastehfar et al., 2020). However, mutations outside the hotspot regions also have been reported (Hou et al., 2019).

Recently, IC with multiple *Candida* species infection has gained increased attention, particularly in patients whose immune systems are compromised, e.g., those living with HIV/AIDS or receiving chemotherapy (García-Salazar et al., 2024; Sadeghi et al., 2024). A comprehensive analysis over 10 years showed that 2.8% of patients had mixed fungemia, indicating the prevalence still remained low (Ferngren et al., 2024). However, mixed candidiasis could complicate diagnosis and treatment, because different *Candida* species might exhibit varying degrees of virulence and resistance to antifungal therapies. According to a previous study (Soriano-Abarca et al., 2024), the overexpression of *HWP1* (encoding virulence factor hyphal wall protein 1) and *ASL3* (encoding virulence factor agglutinin-like sequence 3) under mixed growth conditions of *C. albicans* and non-*C. albicans* indicated a synergistic relationship and increased mycelial growth and adhesion. The interplay between multiple species might lead to more severe infections. Therefore, comprehending the antifungal susceptibility profiles and resistance mechanisms of each type of *Candida* species is of paramount importance for clinical therapy and prognosis of IC.

However, IC caused by multiple *Candida* species is not reported frequently in China. Moreover, the antifungal resistance mechanisms in these pathogens have not been explored in depth and are thus mostly unknown. Herein, we present eight clinical isolates from four different *Candida* species, including the rare *C. norvegensis*, which were isolated from one patient. This complex infection was associated with cross-resistance to azole and echinocandin drugs. Moreover, the molecular mechanisms of the multidrug resistance phenotypes were clarified.

## 2 Materials and methods

### 2.1 Detection and identification of *Candida* isolates

The eight strains were isolated from the infecting mixed fungemia population using single-colony isolation techniques. Briefly, clinical samples were cultured on chromogenic *Candida* agar plates (CHROMagar^™^, Aubervilliers, France) and incubated for 48 h at 37°C. The resultant colonies were preliminarily examined for their colony color following the supplier’s guidelines to identify the *Candida* spp. Then the individual colonies with distinct morphological characteristics were picked and subcultured for purification.

Species identification was further confirmed utilizing internal transcribed spacer (ITS) sequencing and Matrix-assisted laser desorption/ionization time-of-flight mass spectrometry (MALDI-TOF MS) (Vitek MS, bioMérieux, Marcy l’Etoile, France). Primers ITS1 and ITS4 (ITS1:5′-TCCGTAGGTGAACCTGCG-3′; ITS4:5′-TCCTCCGCTTATTGATATGC-3′) were employed to amplify and sequence the ITS regions (Zhang et al., 2014). BLAST searches^1^ were employed for species identification (>99% similarity).

The Ethics Committee of Ruijin Hospital, Shanghai Jiaotong University School of Medicine provided approval for this study (approval number: MX-B4621R).

### 2.2 Testing susceptibility to antifungals

A colorimetric microdilution panel, DL-96Fungus (Dier Biotech, Guangzhou, China) was employed to test the *in vitro* susceptibility of the isolates to eight drugs (amphotericin B, 5-flucytosine, micafungin, caspofungin, fluconazole, voriconazole, posaconazole and itraconazole) according to the supplier’s guidelines. The isolates were grown at 37°C for 24 h to ascertain the minimum inhibitory concentrations (MICs). The quality control species comprised *Candida parapsilosis* ATCC 22019 and *Candida krusei* ATCC 6258. *C. parapsilosis* ATCC 22019 is a well-established QC strain with known susceptibility to azoles and echinocandins, making it suitable for verifying the performance of these antifungals. *C. krusei* ATCC 6258 is intrinsically resistant to fluconazole, serving as a control for azole resistance testing. Resistance breakpoints for *Candida* species (*C. albicans*, *C. glabrata*, and *C. tropicalis*) in this study were defined according to the guidelines of the Clinical and Laboratory Standards Institute (CLSI; M27M44S) (Clinical and Laboratory Standards Institute, 2022b). The MIC values of the two control strains were consistently within the CLSI-defined acceptable ranges for each antifungal agent tested, confirming the validity of our susceptibility results. For those isolates without CLSI-determined clinical breakpoints, the epidemiological cut-off values (ECVs) were used to define the isolates as wild-type (WT) or non-wild-type (non-WT) (Clinical and Laboratory Standards Institute, 2022a). However, because of the scarcity of *C. norvegensis*, there are no clinical breakpoints or ECVs for this species.

### 2.3 PCR amplification and sequence analysis of genes related to antifungal resistance

DNA extraction and purification from overnight suspension were performed employing a TIANamp Yeast DNA Kit (Tiangen Biotech, Beijing, China) in accordance with the supplier’s manual. To identify mutations associated with antifungal resistance in the resistant isolates, the open reading frames (ORFs) of Cg*ERG11*, Cg*PDR1*, Cg*FKS1*, and Cg*FKS2* in *C. glabrata* and *CtERG11* in *C. tropicalis* were amplified using PrimeSTAR^®^ HS DNA Polymerase (Takara, Shiga, Japan) and specific primers (Supplementary Table S1) (Jiang et al., 2013; Wang et al., 2021). Genewiz (Beijing, China) determined the sequences of all the amplicons in both directions. The sequences were compared with those of reference strains and Snapgene 7.2 software (GSL Biotech LLC, Boston, MA, United States) was employed to identify single nucleotide polymorphisms (SNPs). The obtained nucleotide sequences were compared with those deposited in GenBank (*CgERG11*, accession no. L40389.1; *CgPDR1*, accession no. NC005967; *CgFKS1*, accession no. HM366440.1; *CgFKS2*, accession no. HM366442.1; and *CtERG11*. accession no. M23673).

### 2.4 Quantification of rhodamine 6G efflux

The fluorescent dye rhodamine 6G resembles the membrane transporters of azoles in certain *Candida* spp. (Yao et al., 2019). Therefore, rhodamine 6G efflux pump activity of *Candida* spp. was determined utilizing an ultraviolet-visual (UV–Vis) spectrophotometer (Thermo Scientific, Waltham, MA, United States) (Fattouh et al., 2024). Yeast peptone dextrose (YPD) medium was used to adjust overnight-cultured isolates to 5 × 10^7^ cells/mL. The *Candida* cells were centrifuged and rinsed thrice using glucose-free phosphate-buffered saline (PBS). Subsequently, the cells were depleted of energy by incubation for 4 h at 30°C with continuous shaking. Next, we added rhodamine 6G (Sigma-Aldrich, St Louis, MO, United States; final concentration = 10 μM) to the cells and shook them at for 2 h at 30°C. Following three rinses in cold sterile PBS, the *Candida* cells were reconstituted in 10 mL of PBS, followed by incubation at 30°C with gentle shaking. At 15, 30, 45, and 60 min, we took 1 mL of supernatant, which was centrifuged at 9,000 × *g* for 2 min to remove the *Candida* cells. The optical density (OD) values at 527 nm were determined for the supernatant employing a UV–Vis spectrophotometer. We generated a standard curve to determine the concentration of rhodamine 6G in the supernatant. Average values were determined from triplicated experiments. The negative control was treated with sodium azide (NaN₃), an ATPase inhibitor, to block energy-dependent efflux pump activity. A known high-efflux *Candida* strain, which was previously stored in the lab, was used as the positive control.

### 2.5 RNA extraction and quantitative real-time reverse transcription PCR

The isolates were grown in YPD medium to the mid exponential phase [OD at 600 nm (OD_600_) = 0.4–0.5] and then a Yeast RNAiso Reagent Kit (Takara, Shiga, Japan) was used to extract total RNA according to the supplier’s guidelines. Then, a PrimeScript RT reagent kit (Takara) was employed to reverse transcribe RNA to cDNA. Each reaction can reverse transcribe 1 μg RNA. The expression levels of *CgCDR1*, *CgCDR2*, *CgSNQ2*, *CgPDR1*, *CgERG11*, and *CtERG11* were determined utilizing the cDNA as templates and a SYBR Premix Ex Taq Kit (Takara, Shiga, Japan) on a 7,300 real-time PCR System (Applied Biosystems, Shanghai, China), as detailed in a previous publication. The cDNA content in per RT-qPCR reaction is 75 ng/20 μL. The qPCR reactions were carried out thus: 95°C denaturation for 45 s, then 40 cycles of 5 s at 95°C and 45 s at 60°C. Table 1 shows the primers used for qPCR (Jiang et al., 2013; Yao et al., 2019). Target gene quantification employed the 2^−△△CT^ method (Livak and Schmittgen, 2001). The expression of *ACT1* gene (encoding actin1) was detected as an internal control. The RT-qPCR assays were carried out in triplicate. No-template control reactions were performed for each primer set by replacing the cDNA template with nuclease-free water. To rule out genomic DNA contamination, we conducted no-reverse transcription control reactions by omitting the reverse transcriptase enzyme during cDNA synthesis. To confirm the specificity and efficiency of our RT-qPCR assay, positive controls were included for each target gene using corresponding clinical strains with confirmed highly expression of the respective gene.

**Table 1:** Primers used for real-time PCR.

Gene	Sequence (5′-3′)
CgCDR1	F: ACACCAACAACAGCATCT R: ATTCTCCGCTTACCTACG
CgCDR2	F: CAACGCTATGAGGGAAAA R: AACATAAGTGGCGTGGGT
CgSNQ2	F: ACCATGTGTTCTGAATCAATCAAT R: TCGACATCATTACAATACCAGAAA
CgERG11	F: TGGAAGCAGTGAAGATAGT R: AGTGTTCGGTAAAGGTGT
CgPDR1	F: AGCCTTGCCGATAGTCATAC R: AAGGTCAGGGCATACTTCAG
CtERG11	F: CTACTCCCAAAAAAAACCATA R: TAAACCTAATCCCAAGACATC
ACT1	F: AGAAGTTGCTGCTTTAGTT R: GACAGCTTGAATGGAAAC

### 2.6 Whole genome sequencing and bioinformatics analysis

Whole genome sequencing was performed for two *C. glabrata* strains RJ04 and RJ05. The genome DNA was extracted as described previously in section 2.3. Library preparation and next-generation sequencing was conducted by Illumina PE150. Raw sequencing data was assessed for quality using FastQC. Then the short sequencing reads were *de novo* assembled into contiguous sequences (contigs). FastANI v1.34^2^ was applied for fast alignment-free computation of whole-genome Average Nucleotide Identity (ANI). Snippy v4.6.0 was used to detect the Single Nucleotide Polymorphisms (SNPs) based on the whole genome. *C. glabrata* RJ04 was used as reference strain.

### 2.7 Statistical analysis

Data were analyzed and visualized using GraphPad Prism 10 software (GraphPad Inc., La Jolla, CA, United States). Two-way ANOVA was employed to assess statistical difference of extracellular concentrations of rhodamine 6G relative to that in the control. Multiple unpaired *t*-tests were used to test the significance of expression level change. *p* < 0.05 is considered as a significant difference.

## 3 Results

### 3.1 Overview of the clinical isolates

**Figure 1:**
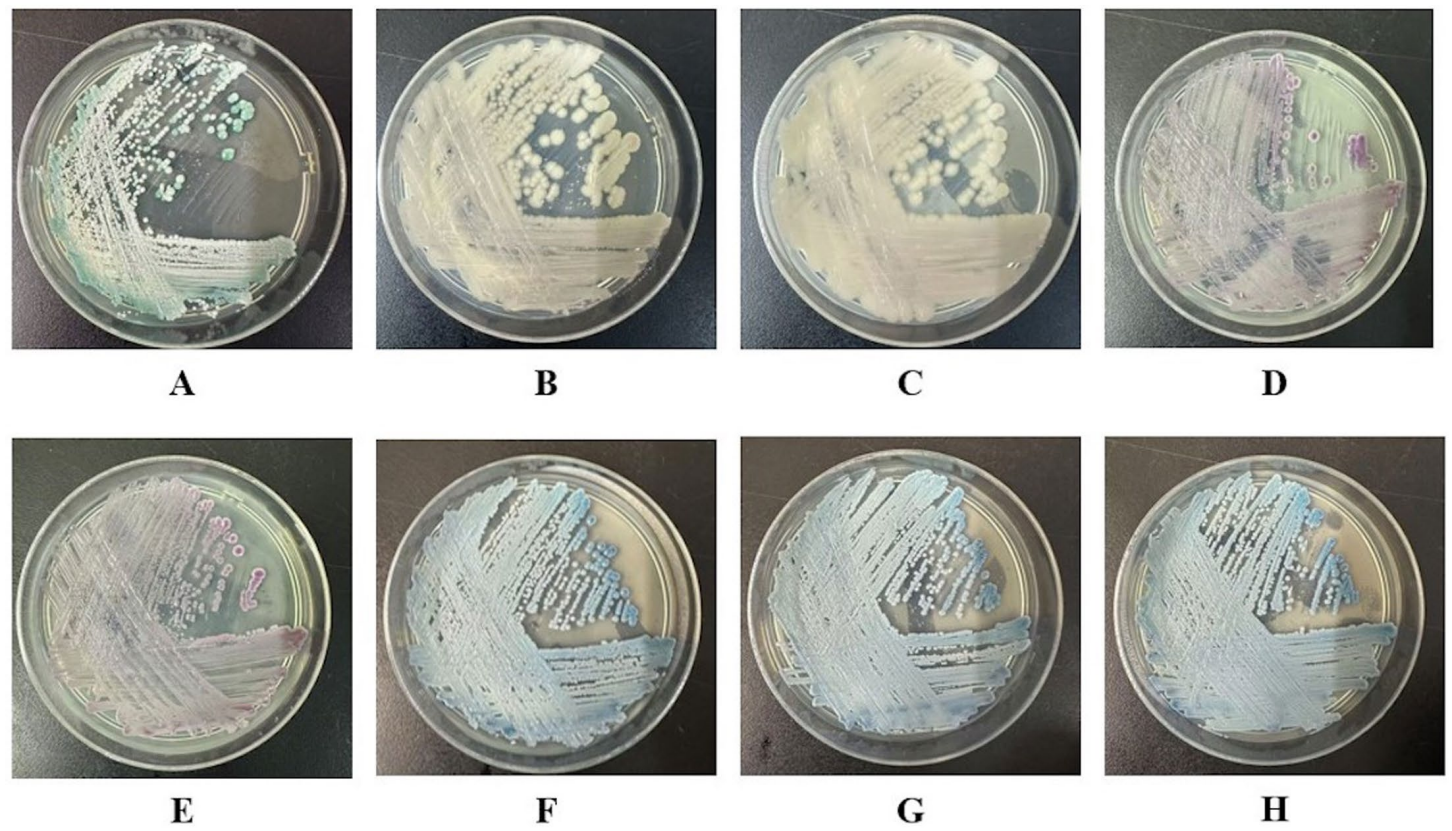
*Candida* colony morphology on the chromogenic *Candida* agar plates (CHROMagar^™^, Aubervilliers, France) of all the eight strains RJ01 ~ RJ08 collected from the same patient. **(A)** *C. albicans* RJ01; **(B,C)** *C. norvegensis* RJ02, RJ03; **(D,E)** *C. glabrata* RJ04, RJ05; **(F–H)** *C. tropicalis* RJ06, RJ07, RJ08.

Figure 1 shows all the *Candida* species isolated from an 86-year-old female patient who was treated at Ruijin Hospital, Shanghai Jiao Tong University School of Medicine. She was admitted to the emergency room suffering from abdominal pain, emesis, and unconsciousness. An abdominal computed tomography scan showed choledocholithiasis with obstructive dilation and pneumatosis. She was then diagnosed with obstructive suppurative cholangitis and transferred to the ICU for further treatment.

**Figure 2:**
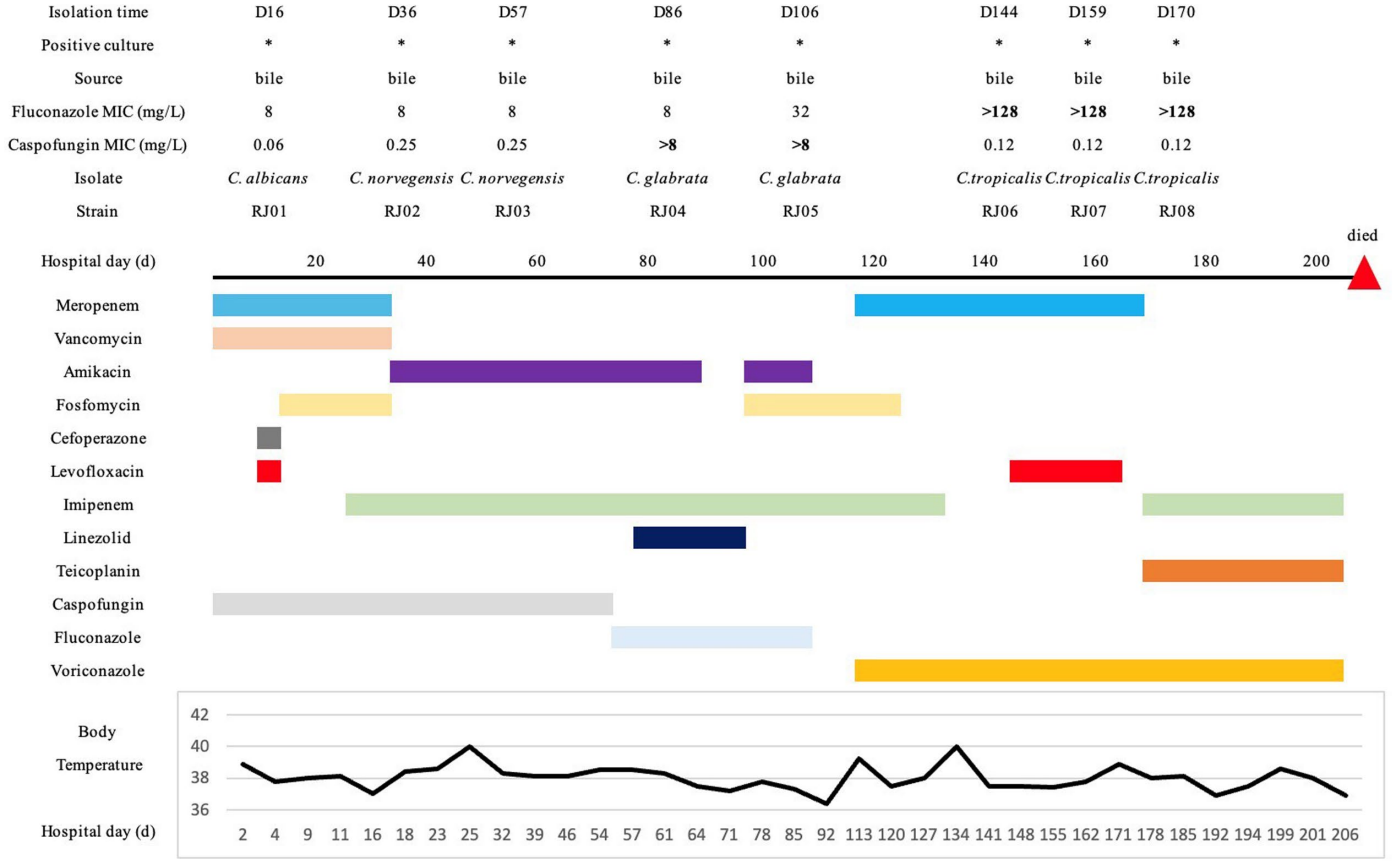
Medical history chart. The specimen sources, drug susceptibility, and isolation times of all the isolates were marked on the chart. All the medication histories and treatment durations were indicated in different colors. Additionally, the temperature changes during hospitalization were displayed at the bottom of the chart.

As can be seen from Figure 2, at the beginning of treatment, *C. albicans* (RJ01) and *C. norvegensis* (RJ02, RJ03) were isolated from bile specimens. The patient was treated with CAS (50 mg qd) and FLU (200 mg qd) successively. After treatment, *C. albicans* and *C. norvegensis* were no longer detected. Instead, *C. glabrata* (RJ04, RJ05) isolates were detected in bile specimens. Then, the treatment switched to VRC (150 mg q12h). During this treatment period, *C. tropicalis* (RJ06, RJ07, RJ08) isolates were consistently detected in bile specimens. The detection of multiple *Candida* species from sterile fluid bile suggested that the patient might have IC. Meanwhile, *Acinetobacter baumannii* was repeatedly detected in her sputum and bile specimens, and later, blood infection with *Staphylococcus epidermidis* also appeared. The patient died after treatment failed because of septic shock caused by a severe infection throughout her body.

### 3.2 Antifungal susceptibility of the *Candida* isolates

Table 2 shows the result of antifungal susceptibility testing of all eight isolates in this study toward the eight tested antifungal drugs. *C. norvegensis* (RJ02, RJ03) showed relatively low MIC values toward most of the antifungal drugs. However, there were no clinical breakpoints or epidemiological cut-off values for *C. norvegensis* because of its rarity. Additionally, *C. albicans* (RJ01) showed susceptibility or non-WT to all the tested drugs. For *C. glabrata*, isolate RJ04 displayed high-level resistance to MIF and CAS (MIC > 8 μg/mL). However, *C. glabrata* RJ05 showed an additional non-WT to azoles (POS and ITR) after treatment (MIC > 16 μg/mL). Moreover, all *C. tropicalis* isolates (RJ06, RJ07, and RJ08) demonstrated significant resistance to FLU (MIC > 128 μg/mL).

**Table 2:** Antifungal susceptibility results of all the eight clinical strains isolated from the patient sequentially.

*Candida* species	Isolate no.	Isolation time	Source	MIC (μg/mL)							
				AMB	FCT	MIF	CAS	FLU	VRC	POS	ITR
*C. albicans*	RJ01	Day 16	bile	0.25	0.25	≤0.008	0.06	8	0.12	0.5	0.5
*C. norvegensis*	RJ02	Day 36	bile	0.5	8	0.03	0.25	8	0.25	0.12	0.25
*C. norvegensis*	RJ03	Day 57	bile	0.25	8	0.03	0.25	8	0.25	0.12	0.25
*C. glabrata*	RJ04	Day 86	bile	0.5	≤0.12	**>8**	**>8**	8	0.12	0.5	0.5
*C. glabrata*	RJ05	Day 106	bile	0.25	≤0.12	**>8**	**>8**	32	2	**>16**	**>16**
*C. tropicalis*	RJ06	Day 144	bile	0.5	0.25	≤0.008	0.12	**>128**	8	0.5	1
*C. tropicalis*	RJ07	Day 159	bile	0.25	0.25	≤0.008	0.12	**>128**	8	0.5	1
*C. tropicalis*	RJ08	Day 170	bile	0.25	0.25	≤0.008	0.12	**>128**	8	0.5	1

MIC values indicating resistance/non-wild-type to antifungal drugs are presented in bold.

MIC, minimum inhibitory concentration; AMB, amphotericin B; FCT, 5-flucytosine; MIF, micafungin; CAS, caspofungin; FLU, fluconazole; VRC, voriconazole; POS, posaconazole; ITR, itraconazole.

### 3.3 Homology analysis of two *Candida glabrata* strains

The two *C. glabrata* strains RJ04 and RJ05 exhibited significant differences in their susceptibility to azoles. Based on whole-genome data, we performed a homology alignment between the two strains. The assembled genomes were subjected to cross-comparison analysis, revealing an overall similarity of 99.9973%. RJ04 and RJ05 were proven to be highly homologous, and might be two mutant strains derived from the same maternal strain. Further, using RJ04 as the reference genome, we identified 14 SNP variants, 3 complex variants, 1 deletion variant, and 1 MNP variant between the two samples at the read level. The detailed mutation sites are listed in Table 3.

**Table 3:** Detailed mutation information between *C. glabrata* RJ04 and RJ05.

Mutation type	REF*	ALT*	type	Effect	Product
snp	G	A	CDS	stop_gained c.1088G > A p.Trp363*	Protein KES1
snp	T	C	–	–	–
del	ATT	A	–	–	–
Complex	TGCGGGGGTGGGAGG	GTCGGAGGTTTGAAT	CDS	missense_variant c.112_126delCCTCCCACCCCCGCAinsATTCAAACCTCCGAC p.ProProThrProAla38IleGlnThrSerAsp	Negative growth regulatory protein NGR1
snp	G	T	CDS	missense_variant c.103C > A p.Pro35Thr	Negative growth regulatory protein NGR1
Complex	TGGG	CGGT	CDS	synonymous_variant c.90_93delCCCAinsACCG p.32	Negative growth regulatory protein NGR1
snp	T	G	–	–	–
snp	G	A	–	–	–
snp	G	T	–	–	–
snp	G	C	–	–	–
snp	T	C	–	–	–
snp	C	G	–	–	–
snp	G	A	CDS	synonymous_variant c.393C > T p.Gly131Gly	Hypothetical protein
Complex	AACCA	CACCG	CDS	missense_variant c.419_423delTGGTTinsCGGTG p.Val140Ala	Flocculation protein FLO9
snp	T	C	CDS	missense_variant c.407A > G p.Gln136Arg	Flocculation protein FLO9
snp	N	G	–	–	–
mnp	NNN	GAA	–	–	–
snp	G	A	–	–	–
snp	A	T	–	–	–

The mutation position, effect, products were listed in the table. *C. glabrata* RJ04 was used as reference.

*REF, Reference; ALT, Alteration.

### 3.4 Efflux of rhodamine 6G

A UV–Vis spectrophotometer was used to determine rhodamine 6G efflux. *C. glabrata* and *C. tropicalis* acquire resistance to azole antifungals via efflux pump overexpression (Jiang et al., 2013; Yao et al., 2019). Rhodamine 6G was introduced into the cells of *C. glabrata* and *C. tropicalis*, and then its efflux into the extracellular fluid of isolates RJ05, RJ06, RJ07, RJ08, and the control strains was quantified in three biological replicates. The controls comprised *C. glabrata* RJC1 and *C. tropicalis* RJC2. They were preserved in our laboratory and were both susceptible and wild-type to all the tested drugs, including azoles.

**Figure 3:**
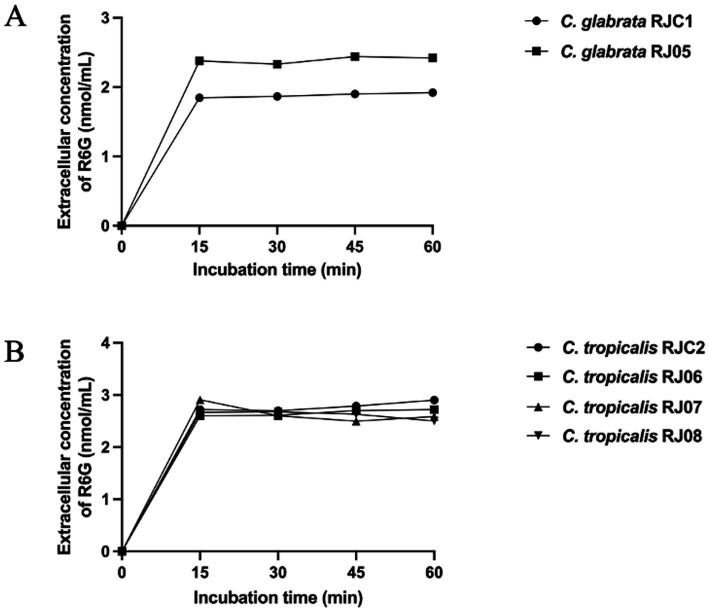
Quantification of extracellular concentration of R6G (nmol/mL) during 60 min. **(A)** Each data point for the control strain *C. glabrata* RJC1 (black circles) and *C. glabrata* RJ05 (black squares). A significant increase in extracellular R6G (*p* = 0.0166) was detected in *C. glabrata* RJ05 compared with the control strain. **(B)** Each data point for the control strain *C. tropicalis* RJC2 (black circles) and test strains *C. tropicalis* RJ06 (black squares), *C. tropicalis* RJ07 (black triangle) and *C. tropicalis* RJ08 (black inverted triangle). No significant increase in extracellular R6G (*p* = 0.3339) was detected in test strains compared with the control strain.

As shown in Figure 3A, at 15, 30, 45, and 60 min, *C. tropicalis* RJ05 exhibited significantly higher extracellular concentrations of rhodamine 6G relative to that in the control. According to two-way ANOVA analysis, the *p*-value was 0.0166, revealing that RJ05 has significantly enhanced efflux activity, thereby contributing to resistance against azole drugs. However, at all timepoints, the concentrations of rhodamine 6G in *C. tropicalis* RJ06, RJ07, and RJ08 were comparable to that of the control strain, and the statistical analysis yielded a *p*-value of 0.3339, indicating that the efflux pump did not contribute to the azole resistance in these *C. tropicalis* isolates (Figure 3B).

### 3.5 Mutations in resistance-related genes *CgERG11*, *CgPDR1*, *CgFKS1*, *CgFKS2*, *CtERG11*

Analysis of the DNA sequence of the *CgERG11* ORFs in *C. glabrata* showed that there were no mutations in this gene in isolates RJ04 and RJ05 in comparison with the *CgERG11* sequence deposited in GenBank (accession number: L40389.1). However, *CgPDR1* in isolate RJ05 encoded a protein with four missense mutations (S76P, V91I, L98S, and T143P). Furthermore, in *C. glabrata* RJ04 and RJ05, a mutation in *FKS1* resulting in an S629P substitution, together with a premature stop codon in *FKS2* (W1497stop), were observed, which mediated high-level resistance to micafungin and caspofungin (MIC > 8 μg/mL). The FKS mutation pattern has not been reported previously (Supplementary Figures S1A–C).

Analysis of the *CtERG11* ORF DNA sequences from *C. tropicalis* RJ06, RJ07, and RJ08 identified two missense mutations, Y132F and S154F (Supplementary Figure S1D).

### 3.6 Expression levels of CgCDR1, CgCDR2, CgSNQ2, CgPDR1, CgERG11 and CtERG11

**Figure 4:**
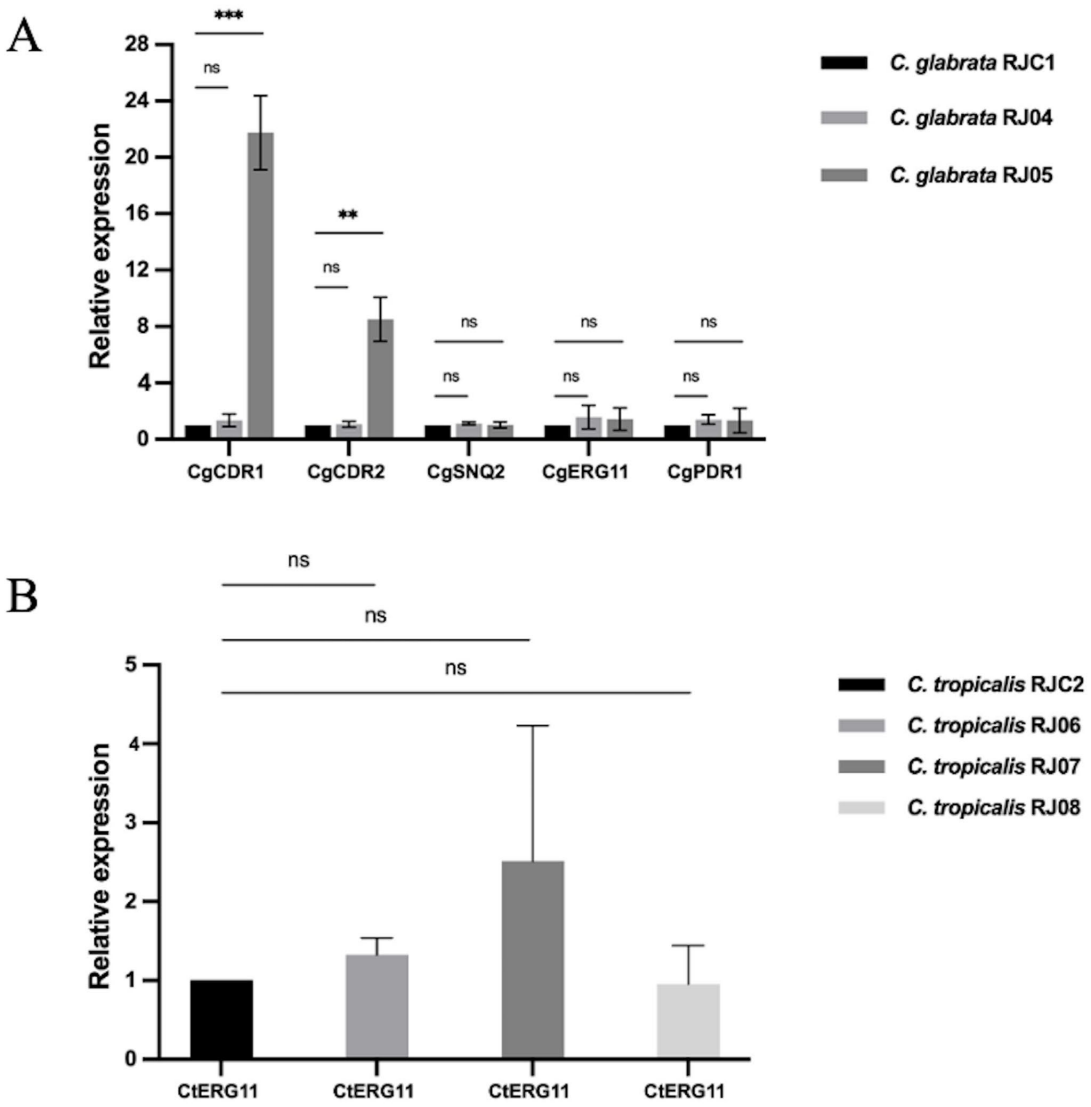
The relative expression level of resistance-related genes. Data presented as means ± SE (error bars) from biological triplicates with technical triplicates. Multiple unpaired t-tests were used to test the significance of expression level change. *p* < 0.05 is considered to indicate a significant difference. **(A)** For *C. glabrata*, the relative expression of each target gene (CgCDR1, CgCDR2, CgSNQ2, CgERG11, CgPDR1) was compared to that of *ACT1* as internal control. *C. glabrata* RJC1 was used as external reference. A significant increase was observed in the CgCDR1 (21.75-fold vs. 1-fold, *p* = 0.000164) and CgCDR2 (8.507-fold vs. 1-fold, *p* = 0.001162) from *C. glabrata* RJ05. No significant increase in the two genes was found in RJ04 (1.346-fold vs. 1-fold, *p* = 0.241881; 1.058-fold vs. 1-fold, *p* = 0.661751). Additionally, no significant change was observed in the CgSNQ2 (1.118-fold vs. 1-fold, *p* = 0.131953; 1.016-fold vs. 1-fold, *p* = 0.899801), CgERG11 (1.575-fold vs. 1-fold, *p* = 0.304047; 1.435-fold vs. 1-fold, *p* = 0.391636), CgPDR1 (1.393-fold vs. 1-fold, *p* = 0.114514; 1.33-fold vs. 1-fold; *p* = 0.552051). **(B)** For *C. tropicalis*, the relative expression of target gene CtERG11 was comparable to that of *ACT1* as internal control. *C. tropicalis* RJC2 was used as external reference. No significant increase was observed in *C. tropicalis* RJ06, RJ07 and RJ08 (1.33-fold vs. 1-fold, *p* = 0.1149; 2.52-fold vs. 1-fold, *p* = 0.2670; 0.95-fold vs. 1-fold, *p* = 0.8875). **p < 0.01, ***p < 0.001, “ns” denotes no significance.

According to the results of RT-qPCR in Figure 4A, CgCDR1 (21.75-fold vs. 1-fold, *p* = 0.000164) and CgCDR2 (8.507-fold vs. 1-fold, *p* = 0.001162) expression levels were significantly higher in *C. glabrata* RJ05 than in the control strain *C. glabrata* RJC1, while *C. glabrata* RJ04 showed no significant difference (*p* > 0.05). In addition, the expression levels of CgSNQ2, CgERG11, and CgPDR1 in *C. glabrata* RJ04 and RJ05 exhibited no significant difference to those in the control isolate (*p* > 0.05). Moreover, the CtERG11 expression levels in three *C. tropicalis* isolates resistant to fluconazole (RJ06, RJ07, RJ08) were not increased significantly compared with those in *C. tropicalis* RJC2 (*p* > 0.05) (Figure 4B).

## 4 Discussion

IC caused by multiple *Candida* species has attracted the attention of epidemiologists in recent years due to its high mortality rate. Relevant reports remain scarce in China. Besides, clarifying the antifungal drug resistance mechanism of each *Candida* strain is of crucial significance for clinical treatment.

In this work, we revealed a rare clinical case of IC caused by multiple *Candida* species in a patient with obstructive suppurative cholangitis. In the ICU, patients with IC might present as deep-seated candidiasis with candidemia, deep-seated candidiasis or candidemia (Azim and Ahmed, 2024). For deep-seated candidiasis, the most frequently encountered form is intra-abdominal candidiasis (Vergidis et al., 2016). In this study, all the strains were isolated from bile samples from a sterile site, thus meeting the diagnostic criteria. The known risk factors for IC include ICU admission, placement of central venous catheters, treatment with broad-spectrum antibiotics, abdominal surgery, and immunosuppression (Lass-Flörl et al., 2024), which were consistent with the patient’s medical history.

Low susceptibility to certain antifungal drugs is a significant and pressing issue to be resolved for the treatment of IC with multiple *Candida* species. Notably, in mixed infections, different resistant phenotypes of *Candida* species could complement each other, ultimately leading to multidrug resistance. In *C. glabrata*, azole resistance is primarily driven by ATP-binding cassette (ABC) family multidrug transporter overexpression, including that of CDR1, CDR2, and SNQ2 (Bennett et al., 2004; Torelli et al., 2008), which is in accordance with the resistance mechanism in *C. glabrata* RJ05 in this study. The azole resistance was mediated by complex mechanisms comprising increased efflux ability, PDR1 mutation, and increased expression of CDR1 and CDR2. Notably, azole resistance was not likely to be mediated by the substitutions in PDR1 (S76P, V91I, L98S, and T143P), because the mutations were not gain-of-function mutations, as reported previously (Hou et al., 2018). Moreover, antifungal resistance can be acquired by *C. glabrata* during therapy (Borst et al., 2005). In this study, *C. glabrata* RJ05 exhibited non-WT to posaconazole and itraconazole after *in vivo* fluconazole exposure. Echinocandin resistance in *C. glabrata* has been associated with mutations in FKS1 and FKS2 (Pfaller et al., 2012). Mutations in FKS2 are more common than the ones in FKS1 (Zimbeck et al., 2010; Alexander et al., 2013). However, a novel mutational pattern mediating high level echinocandin-resistance (MIC > 8 μg/mL) was found in this study. The mutations were both identified in *C. glabrata* RJ04 and RJ05 strains: an S629P mutation in FKS1 HS1 region and a premature stop codon (W1497stop) in FKS2, which have not been reported previously. Similarly, Hou et al. (2019) has demonstrated that both mutations in FKS1 (W508stop) and FKS2 (E655K) might lead to higher MICs to echinocandins than single mutations. This revealed that more than one mutation within the same isolates may potentially mediate a high-level echinocandin-resistant phenotype in *C. glabrata*. However, more experiments are required to validate this hypothesis. As for *C. tropicalis*, the azole antifungal target cytochrome P450 lanosterol 14a-demethylase encoded by ERG11 play a rather vital role in azole resistance. Two mutations, Y132F and S154F in ERG11, were demonstrated to be responsible for fluconazole resistance in *C. tropicalis* RJ06, RJ07, and RJ08 in this study. This resistance mechanism has been experimentally proven in the previous study and interestingly, these two mutations always occurred together (Jiang et al., 2013).

*In vivo* evolution appears to be quite interesting. Homology analysis showed that RJ05 has 19 SNPs compared to RJ04, suggesting that these two strains are homologous. Previous literature (Misas et al., 2024) has reported that a threshold of 96 SNPs is considered indicative of homology between two strains. Therefore, RJ05 may have evolved from RJ04 under the sustained pressure of fluconazole. In fact, there have been previous reports of *in vivo* evolution of *C. glabrata* resistance to fluconazole, mainly involving the upregulation of CgCDR1, CgCDR2, and CgERG11 expression (Redding et al., 2003). However, in our study, RJ05 exhibits simultaneous resistance to both echinocandins and azoles, which complicates clinical treatment. It is noteworthy that RJ05 has multiple protein mutations compared to RJ04, including in proteins such as KES1, Negative Growth Regulatory Protein NGR1, and Flocculation Protein FLO9. Whether these proteins play a role in the development of azole resistance remains to be further investigated.

*C. norvegensis* is an uncommon *Candida* species. Recently, invasive infections by rare *Candida* species have been on the rise (Pinho et al., 2024). A recent report showed that *C. norvegensis* could display decreased anti-fungal susceptibility (Stavrou et al., 2020). In the clinic, the interpretation of symptoms and options for treatment for uncommon *Candida* spp. are a challenge because we lack clinical breakpoints to determine their susceptibility profile.

Thus, mixed candidiasis poses significant challenges for clinical treatment, especially regarding uncommon *Candida* species, because of clinicians’ lack of experience of these species. An observational cohort study in Europe showed that the decreased attributable mortality might be the result of improved outcomes for bloodstream infections involving *C. parapsilosis* and *C. albicans*, while bloodstream infections involving other *Candida* species still exhibit higher attributable mortality rates (Salmanton-García et al., 2024). Most significantly, mixed candidiasis mediates both azole and echinocandin resistance, resulting in a situation where no effective drugs are available. This also encourages the development of new antifungal drugs.

In summary, this study aids the clinical treatment of IC and deepens our knowledge regarding the mechanisms underlying *Candida* resistance to multiple drugs. Although multiple fungal infections are still relatively rare (Ferngren et al., 2024), the resulting multi-drug resistance and pathogenicity should be taken seriously. Future studies involving mixed populations from multiple patients will be necessary to validate our observations and provide a broader perspective on the diversity and clinical significance of mixed candidiasis. An in-depth comprehension of the dynamics of these co-infections, including their epidemiology, risk factors, and underlying mechanisms is essential to develop targeted treatment strategies and improve patient management in clinical settings.

## Data Availability

The datasets presented in this study can be found in online repositories. The names of the repository/repositories and accession number(s) can be found in the article/Supplementary material.

## Footnotes

^1^ https://blast.ncbi.nlm.nih.gov/Blast.cgi

^2^ https://github.com/ParBLiSS/FastANI
