# Supplementary material for: Emergence of invasive candidiasis with multiple Candida species exhibiting azole and echinocandin resistance
Source: Front Microbiol. 2025 Mar 25;16:1550894. doi: 10.3389/fmicb.2025.1550894 (PMC11975943; doi:10.3389/fmicb.2025.1550894)
Supplement: Supplementary file 1 [file Data_Sheet_1.pdf]

A

B

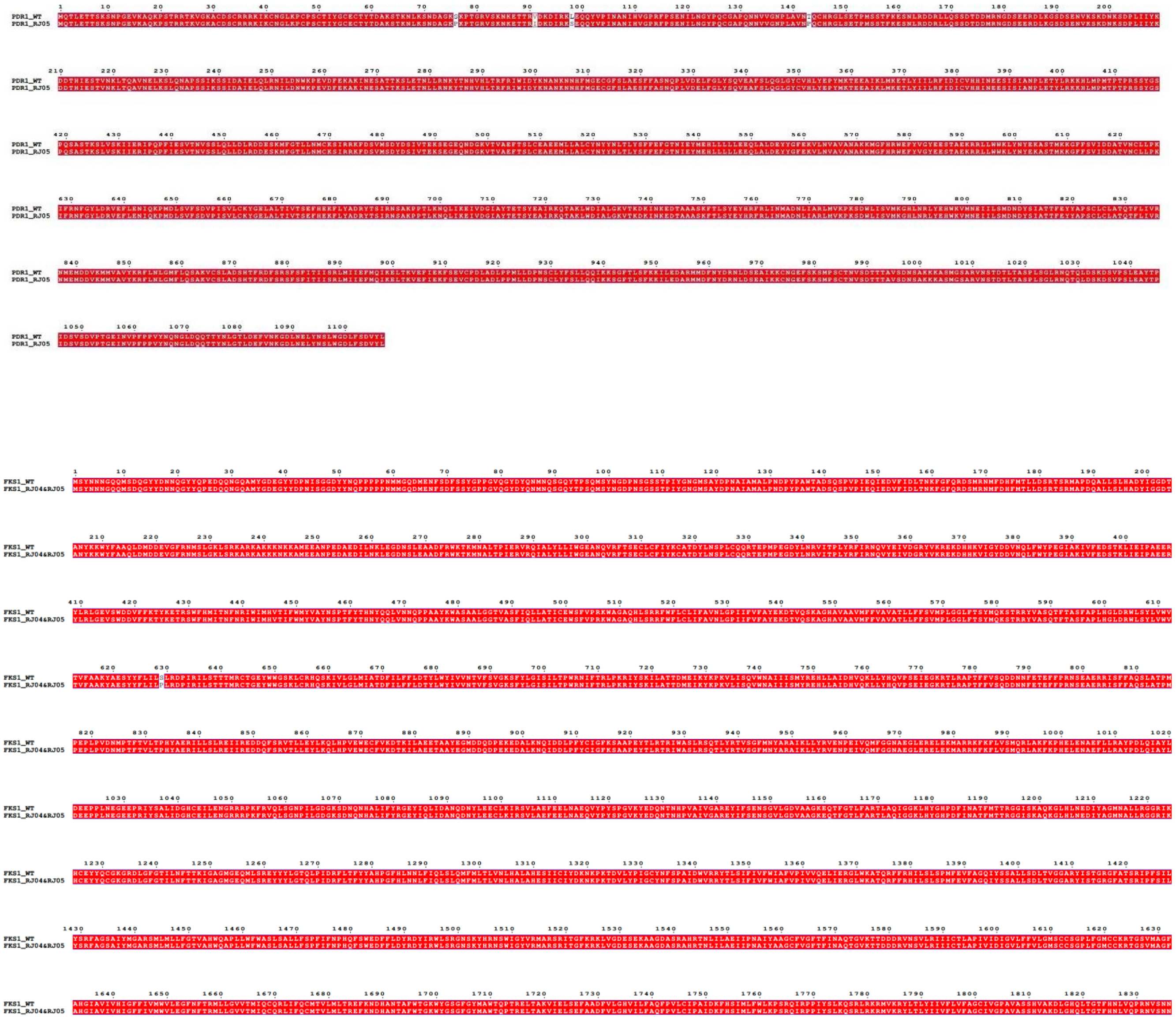

C

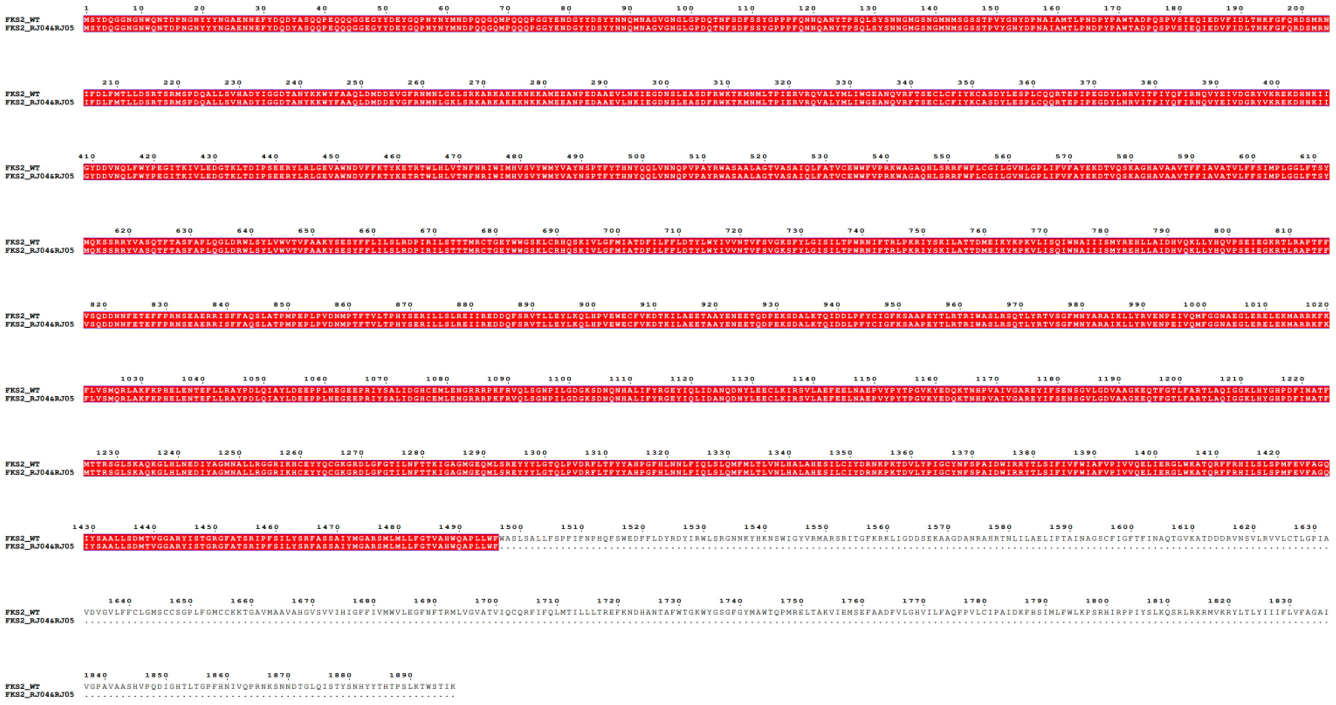

D

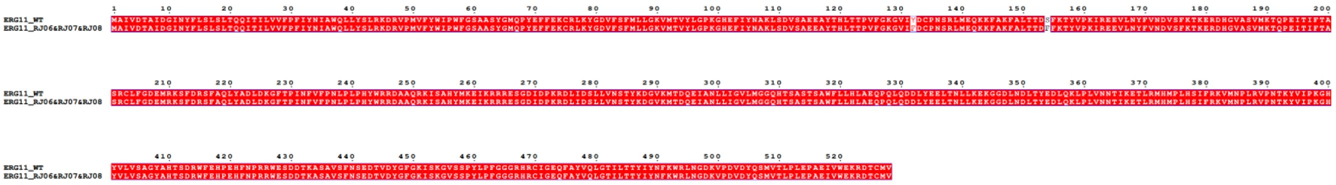

Supplementary Figure S1. Protein sequence alignment. (A): Four missense mutations S76P, V91I, L98S and T143P in PDR1 were detected in *C. glabrata* RJ05. (B): Missense mutation S629P in FKS1 was found in *C. glabrata* RJ04 and RJ05. (C): A premature stop codon (W1497stop) in FKS2 were discovered in *C. glabrata* RJ04 and RJ05. (D): Two missense mutations Y132F and S154F in ERG11 were found in ERG11 in *C. tropicalis* RJ06, RJ07 and RJ08.
