# Supplementary material for: Emergence of invasive candidiasis with multiple Candida species exhibiting azole and echinocandin resistance
Source: Front Microbiol. 2025 Mar 25;16:1550894. doi: 10.3389/fmicb.2025.1550894 (PMC11975943; doi:10.3389/fmicb.2025.1550894)
Supplement: Supplementary file 2 [file Table_1.docx]

**Supplementary Table S1.** Primers used for PCR and sequencing in this study.

| **Primer** | Sequence (5’—3’) |
| --- | --- |
| CgERG11-1 | F: ACAATAACATGTCCACTGAAAACAC  R: AGCTTGTTGGGCATGATCTCT |
| CgERG11-2 | F: GGACAAGGGTTTCACCCCAA  R: TTAGCAGGGGCAGTTGGTAG |
| CgPDR1-1 | F: TCGTATTGCCATTGTGATATGGA  R: TGAGTTCATTAACTGCCTGTGT |
| CgPDR1-2 | F: ATGAGGAATGGTGACTCGGA  R: GCTCTTTGTACTAGCACTTTGTGG |
| CgPDR1-3 | F: TCGATTGCCAACCCGTTAGA  R: ACAGTTGACAGTAGCATCATCA |
| CgPDR1-4 | F: CCGTTGGGAGTTTTACGTCG  R: CTGCGTAGCTAAACACAGACA |
| CgPDR1-5 | F: TGGTGAAACCAAAATCAGATTGGC  R: AAGCCCGATAAGGGAGATGC |
| CgPDR1-6 | F: TGGACTTCAATTACGACCGCA  R: TCGCTAATTTGAGGTAGTCTAAGT |
| CgFKS1-1 | F: ATGTCTTACAATAATAACGGAC  R: ATAGCGATGGCATTAGGATC |
| CgFKS1-2 | F: ATAGCGATGGCATTAGGATC  R: GAAGTCGGTTTCAGAAACAT |
| CgFKS1-3 | F: GAAGTCGGTTTCAGAAACAT  R: TACACACACAACTATCAACA |
| CgFKS1-4 | F: TACACACACAACTATCAACA  R: TTGGCTACTACTGATATGGA |
| CgFKS1-5 | F: TTGGCTACTACTGATATGGA  GAACTGGAAAACGCTGAATT |
| CgFKS1-6 | F: GAACTGGAAAACGCTGAATT  R: GTGAACAAATGTTGTCCCGT |
| CgFKS1-7 | F: GTGAACAAATGTTGTCCCGT  R: GCCCAAACAGGTGTTAAGAC |
| CgFKS1-8 | F: GCCCAAACAGGTGTTAAGAC  R: TTATTTGATTGTAGACCAGGTC |
| CgFKS2-1 | F: ATGTCTTACGATCAAGGTGG  R: ACCGTTGCTGCCCATCCCAT |
| CgFKS2-2 | F: ACCGTTGCTGCCCATCCCAT  R: GGTATTTCGCAGCTCAGCTT |
| CgFKS2-3 | F: GGTATTTCGCAGCTCAGCTT  R: ACTGGTAAACAATCAACCTG |
| CgFKS2-4 | F: ACTGGTAAACAATCAACCTG  R: TGCTATCGACCATGTTCAGA |
| CgFKS2-5 | F: TGCTATCGACCATGTTCAGA  R: TGGATGAAGAACCTCCTTTG |
| CgFKS2-6 | F: TGGATGAAGAACCTCCTTTG  R: GAACAAATGTTGTCCCGTGA |
| CgFKS2-7 | F: GAACAAATGTTGTCCCGTGA  R: GCAGGTAGTTGTTTCATTGG |
| CgFKS2-8 | F: GCAGGTAGTTGTTTCATTGG  R: TTATTTTATAGTGGACCAGGTCTT |
| CtERG11-1 | F: TCTGACATGGTGTGTGTGTG  R: ATTGATGCCATCAATGGCAG |
| CtERG11-2 | F: ATCCCACAGGCTTATTTGAAA  R: GGTCTCTTTCCTTGGTTTTG |
| CtERG11-3 | F: TGCTGAAGAAGCTTATACCC  R: CAAGGAATCAATCAAATCTCTC |
| CtERG11-4 | F: GGTGGTCAACATACTTCTGC  R: AGCAGGTTCTAATGGTAAGG |
| CtERG11-5 | F: AAACGGTGATAAGGTTCCAG  R: TCCCAAGACATCAAACCCTG |
